# Major Genetic Risk Factors for Dupuytren's Disease Are Inherited From Neandertals

**DOI:** 10.1093/molbev/msad130

**Published:** 2023-06-14

**Authors:** Richard Ågren, Snehal Patil, Xiang Zhou, FinnGen., Aarno Palotie, Mark Daly, Bridget Riley-Gills, Howard Jacob, Dirk Paul, Athena Matakidou, Adam Platt, Heiko Runz, Sally John, George Okafo, Nathan Lawless, Robert Plenge, Joseph Maranville, Mark McCarthy, Julie Hunkapiller, Margaret G Ehm, Kirsi Auro, Simonne Longerich, Caroline Fox, Anders Mälarstig, Katherine Klinger, Deepak Raipal, Eric Green, Robert Graham, Robert Yang, Chris ÓDonnell, Tomi Mäkelä, Jaakko Kaprio, Petri Virolainen, Antti Hakanen, Terhi Kilpi, Markus Perola, Jukka Partanen, Anne Pitkäranta, Juhani Junttila, Raisa Serpi, Tarja Laitinen, Veli-Matti Kosma, Jari Laukkanen, Marco Hautalahti, Outi Tuovila, Raimo Pakkanen, Jeffrey Waring, Bridget Riley-Gillis, Fedik Rahimov, Ioanna Tachmazidou, Chia-Yen Chen, Heiko Runz, Zhihao Ding, Marc Jung, Shameek Biswas, Rion Pendergrass, Julie Hunkapiller, Margaret G Ehm, David Pulford, Neha Raghavan, Adriana Huertas-Vazquez, Jae-Hoon Sul, Anders Mälarstig, Xinli Hu, Katherine Klinger, Robert Graham, Eric Green, Sahar Mozaffari, Dawn Waterworth, Nicole Renaud, Máen Obeidat, Samuli Ripatti, Johanna Schleutker, Markus Perola, Mikko Arvas, Olli Carpén, Reetta Hinttala, Johannes Kettunen, Arto Mannermaa, Katriina Aalto-Setälä, Mika Kähönen, Jari Laukkanen, Johanna Mäkelä, Reetta Kälviäinen, Valtteri Julkunen, Hilkka Soininen, Anne Remes, Mikko Hiltunen, Jukka Peltola, Minna Raivio, Pentti Tienari, Juha Rinne, Roosa Kallionpää, Juulia Partanen, Ali Abbasi, Adam Ziemann, Nizar Smaoui, Anne Lehtonen, Susan Eaton, Heiko Runz, Sanni Lahdenperä, Shameek Biswas, Julie Hunkapiller, Natalie Bowers, Edmond Teng, Rion Pendergrass, Fanli Xu, David Pulford, Kirsi Auro, Laura Addis, John Eicher, Qingqin S Li, Karen He, Ekaterina Khramtsova, Neha Raghavan, Martti Färkkilä, Jukka Koskela, Sampsa Pikkarainen, Airi Jussila, Katri Kaukinen, Timo Blomster, Mikko Kiviniemi, Markku Voutilainen, Mark Daly, Ali Abbasi, Jeffrey Waring, Nizar Smaoui, Fedik Rahimov, Anne Lehtonen, Tim Lu, Natalie Bowers, Rion Pendergrass, Linda McCarthy, Amy Hart, Meijian Guan, Jason Miller, Kirsi Kalpala, Melissa Miller, Xinli Hu, Kari Eklund, Antti Palomäki, Pia Isomäki, Laura Pirilä, Oili Kaipiainen-Seppänen, Johanna Huhtakangas, Nina Mars, Ali Abbasi, Jeffrey Waring, Fedik Rahimov, Apinya Lertratanakul, Nizar Smaoui, Anne Lehtonen, David Close, Marla Hochfeld, Natalie Bowers, Rion Pendergrass, Jorge Esparza Gordillo, Kirsi Auro, Dawn Waterworth, Fabiana Farias, Kirsi Kalpala, Nan Bing, Xinli Hu, Tarja Laitinen, Margit Pelkonen, Paula Kauppi, Hannu Kankaanranta, Terttu Harju, Riitta Lahesmaa, Nizar Smaoui, Alex Mackay, Glenda Lassi, Susan Eaton, Hubert Chen, Rion Pendergrass, Natalie Bowers, Joanna Betts, Kirsi Auro, Rajashree Mishra, Majd Mouded, Debby Ngo, Teemu Niiranen, Felix Vaura, Veikko Salomaa, Kaj Metsärinne, Jenni Aittokallio, Mika Kähönen, Jussi Hernesniemi, Daniel Gordin, Juha Sinisalo, Marja-Riitta Taskinen, Tiinamaija Tuomi, Timo Hiltunen, Jari Laukkanen, Amanda Elliott, Mary Pat Reeve, Sanni Ruotsalainen, Benjamin Challis, Dirk Paul, Julie Hunkapiller, Natalie Bowers, Rion Pendergrass, Audrey Chu, Kirsi Auro, Dermot Reilly, Mike Mendelson, Jaakko Parkkinen, Melissa Miller, Tuomo Meretoja, Heikki Joensuu, Olli Carpén, Johanna Mattson, Eveliina Salminen, Annika Auranen, Peeter Karihtala, Päivi Auvinen, Klaus Elenius, Johanna Schleutker, Esa Pitkänen, Nina Mars, Mark Daly, Relja Popovic, Jeffrey Waring, Bridget Riley-Gillis, Anne Lehtonen, Jennifer Schutzman, Julie Hunkapiller, Natalie Bowers, Rion Pendergrass, Diptee Kulkarni, Kirsi Auro, Alessandro Porello, Andrey Loboda, Heli Lehtonen, Stefan McDonough, Sauli Vuoti, Kai Kaarniranta, Joni A Turunen, Terhi Ollila, Hannu Uusitalo, Juha Karjalainen, Esa Pitkänen, Mengzhen Liu, Heiko Runz, Stephanie Loomis, Erich Strauss, Natalie Bowers, Hao Chen, Rion Pendergrass, Kaisa Tasanen, Laura Huilaja, Katariina Hannula-Jouppi, Teea Salmi, Sirkku Peltonen, Leena Koulu, Nizar Smaoui, Fedik Rahimov, Anne Lehtonen, David Choy, Rion Pendergrass, Dawn Waterworth, Kirsi Kalpala, Ying Wu, Pirkko Pussinen, Aino Salminen, Tuula Salo, David Rice, Pekka Nieminen, Ulla Palotie, Maria Siponen, Liisa Suominen, Päivi Mäntylä, Ulvi Gursoy, Vuokko Anttonen, Kirsi Sipilä, Rion Pendergrass, Hannele Laivuori, Venla Kurra, Laura Kotaniemi-Talonen, Oskari Heikinheimo, Ilkka Kalliala, Lauri Aaltonen, Varpu Jokimaa, Johannes Kettunen, Marja Vääräsmäki, Outi Uimari, Laure Morin-Papunen, Maarit Niinimäki, Terhi Piltonen, Katja Kivinen, Elisabeth Widen, Taru Tukiainen, Mary Pat Reeve, Mark Daly, Niko Välimäki, Eija Laakkonen, Jaakko Tyrmi, Heidi Silven, Eeva Sliz, Riikka Arffman, Susanna Savukoski, Triin Laisk, Natalia Pujol, Mengzhen Liu, Bridget Riley-Gillis, Rion Pendergrass, Janet Kumar, Kirsi Auro, Iiris Hovatta, Chia-Yen Chen, Erkki Isometsä, Kumar Veerapen, Hanna Ollila, Jaana Suvisaari, Thomas Damm Als, Antti Mäkitie, Argyro Bizaki-Vallaskangas, Sanna Toppila-Salmi, Tytti Willberg, Elmo Saarentaus, Antti Aarnisalo, Eveliina Salminen, Elisa Rahikkala, Johannes Kettunen, Kristiina Aittomäki, Fredrik Åberg, Mitja Kurki, Samuli Ripatti, Mark Daly, Juha Karjalainen, Aki Havulinna, Juha Mehtonen, Priit Palta, Shabbeer Hassan, Pietro Della Briotta Parolo, Wei Zhou, Mutaamba Maasha, Kumar Veerapen, Shabbeer Hassan, Susanna Lemmelä, Manuel Rivas, Mari E Niemi, Aarno Palotie, Aoxing Liu, Arto Lehisto, Andrea Ganna, Vincent Llorens, Hannele Laivuori, Taru Tukiainen, Mary Pat Reeve, Henrike Heyne, Nina Mars, Joel Rämö, Elmo Saarentaus, Hanna Ollila, Rodos Rodosthenous, Satu Strausz, Tuula Palotie, Kimmo Palin, Javier Garcia-Tabuenca, Harri Siirtola, Tuomo Kiiskinen, Jiwoo Lee, Kristin Tsuo, Amanda Elliott, Kati Kristiansson, Mikko Arvas, Kati Hyvärinen, Jarmo Ritari, Olli Carpén, Johannes Kettunen, Katri Pylkäs, Eeva Sliz, Minna Karjalainen, Tuomo Mantere, Eeva Kangasniemi, Sami Heikkinen, Arto Mannermaa, Eija Laakkonen, Nina Pitkänen, Samuel Lessard, Clément Chatelain, Perttu Terho, Sirpa Soini, Jukka Partanen, Eero Punkka, Raisa Serpi, Sanna Siltanen, Veli-Matti Kosma, Teijo Kuopio, Anu Jalanko, Huei-Yi Shen, Risto Kajanne, Mervi Aavikko, Mitja Kurki, Juha Karjalainen, Pietro Della Briotta Parolo, Arto Lehisto, Juha Mehtonen, Wei Zhou, Masahiro Kanai, Mutaamba Maasha, Kumar Veerapen, Hannele Laivuori, Aki Havulinna, Susanna Lemmelä, Tuomo Kiiskinen, L Elisa Lahtela, Mari Kaunisto, Elina Kilpeläinen, Timo P Sipilä, Oluwaseun Alexander Dada, Awaisa Ghazal, Anastasia Kytölä, Rigbe Weldatsadik, Kati Donner, Timo P Sipilä, Anu Loukola, Päivi Laiho, Tuuli Sistonen, Essi Kaiharju, Markku Laukkanen, Elina Järvensivu, Sini Lähteenmäki, Lotta Männikkö, Regis Wong, Auli Toivola, Minna Brunfeldt, Hannele Mattsson, Kati Kristiansson, Susanna Lemmelä, Sami Koskelainen, Tero Hiekkalinna, Teemu Paajanen, Priit Palta, Kalle Pärn, Mart Kals, Shuang Luo, Vishal Sinha, Tarja Laitinen, Mary Pat Reeve, Marianna Niemi, Kumar Veerapen, Harri Siirtola, Javier Gracia-Tabuenca, Mika Helminen, Tiina Luukkaala, Iida Vähätalo, Jyrki Pitkänen, Marco Hautalahti, Johanna Mäkelä, Sarah Smith, Tom Southerington, Kristoffer Sahlholm, Svante Pääbo, Hugo Zeberg

**Affiliations:** Department of Physiology and Pharmacology, Karolinska Institutet, Stockholm, Sweden; Department of Biostatistics, University of Michigan, Ann Arbor, MI, USA; Department of Biostatistics, University of Michigan, Ann Arbor, MI, USA; Department of Physiology and Pharmacology, Karolinska Institutet, Stockholm, Sweden; Department of Integrative Medical Biology, Wallenberg Centre for Molecular Medicine, Umeå University, Umeå, Sweden; Department of Evolutionary Genetics, Max Planck Institute for Evolutionary Anthropology, Leipzig, Germany; Human Evolutionary Genomics Unit, Okinawa Institute of Science and Technology, Okinawa, Japan; Department of Physiology and Pharmacology, Karolinska Institutet, Stockholm, Sweden; Department of Evolutionary Genetics, Max Planck Institute for Evolutionary Anthropology, Leipzig, Germany; Human Evolutionary Genomics Unit, Okinawa Institute of Science and Technology, Okinawa, Japan

**Keywords:** Dupuytren's disease, Neandertals, genome-wide association studies, EPDR1, risk variant, splicing quantitative trait loci

## Abstract

Dupuytren's disease is characterized by fingers becoming permanently bent in a flexed position. Whereas people of African ancestry are rarely afflicted by Dupuytren's disease, up to ∼30% of men over 60 years suffer from this condition in northern Europe. Here, we meta-analyze 3 biobanks comprising 7,871 cases and 645,880 controls and find 61 genome-wide significant variants associated with Dupuytren's disease. We show that 3 of the 61 loci harbor alleles of Neandertal origin, including the second and third most strongly associated ones (*P* = 6.4 × 10^−132^ and *P* = 9.2 × 10^−69^, respectively). For the most strongly associated Neandertal variant, we identify *EPDR1* as the causal gene. Dupuytren's disease is an example of how admixture with Neandertals has shaped regional differences in disease prevalence.

## Introduction

Dupuytren's disease is a fibroproliferative disorder affecting the hand. Initially, nodules develop in the palmar fascia and thicken to form cords, leading to contractures causing fingers to become permanently bent in a flexed position. Although the condition can affect any finger, the ring and middle fingers are most often afflicted (Dutta et al. 2020). Several risk factors have been identified, including age, alcohol consumption, diabetes mellitus, and genetic predisposition (Rydberg et al. 2021). A Danish twin study reported 80% heritability (Larsen et al. 2015), indicating a strong genetic influence. One study estimated the prevalence of Dupuytren's disease among Norwegian individuals over 60 years to be ∼30% (Burge 1999). In contrast, Dupuytren's disease is less prevalent among individuals of primarily African descent. The geographic distribution has given Dupuytren's disease the nickname “Viking disease,” although this designation has been opposed (Ng et al. 2019).

In addition to case reports of Dupuytren's disease in Africa, one study measured the prevalence in a comparable fashion between different ancestries as a part of the Million Veteran Program (Saboeiro et al. 2000). In this study, the prevalence was 0.73% (95% confidence interval [CI]: 0.72–0.75%) among individuals of primarily European descent and 0.13% (95% CI: 0.12–0.14%) among individuals of primarily African descent. We note that a recent study (Salari et al. 2020), that suggested a high African prevalence, erroneously assigned diabetic patients from Jordan (Mustafa et al. 2016) and a small cohort of admixed individuals inhabiting the remote island Tristan da Cunha (Beighton and Valkenburg 1974), as individuals of African ancestry.

Genome-wide association studies (GWASs) have identified 36 risk variants for Dupuytren's disease, several of which are located in the proximity of genes encoding members of the profibrotic Wnt signaling pathway (Dolmans et al. 2011, ten Dam et al. 2016). The very high prevalence of Dupuytren's disease in certain populations, as noted above, indicates that common genetic variants may be powerful modulators of disease risk. Here, we perform a meta-analysis incorporating data from 3 large-scale biobanks, including 7,871 individuals with Dupuytren's disease and 645,880 controls, and identify 21 novel genetic risk variants. The rarity of the disease in African populations led us to investigate if any of the common variants are inherited from Neandertals, given that gene flow from Neandertals is limited in individuals of African ancestry (Chen et al. 2020). We find 3 risk factors for Dupuytren's disease inherited from Neandertals, 2 of which are the second and third major risk factors.

## Results

### A Meta-analysis of Dupuytren's Disease Among 653,741 Europeans

**Fig. 1.**
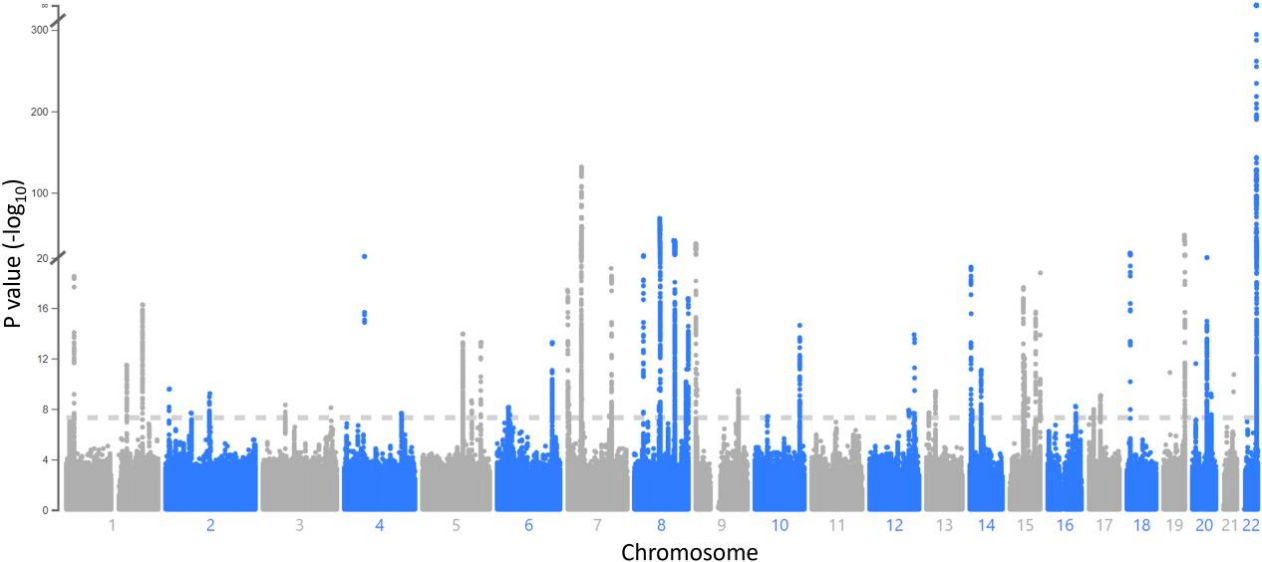
Genome-wide associations with Dupuytren's disease in 7,871 cases and 645,880 controls. Meta-analysis of UK Biobank, FinnGen, and MGI. The dotted horizontal line represents the genome-wide significance threshold (*P* = 5 × 10^−8^). Coordinates are in *hg38*. See supplementary table S1, Supplementary Material online for genome-wide significant loci.

We used 3 biobanks with individuals of primarily European descent, the UK Biobank (3,488 cases, 377,173 controls), FinnGen R7 (4,051 cases and 231,460 controls), and Michigan Genomics Initiative (MGI) freeze 3 (332 cases and 37,247 controls), to identify genetic risk variants for Dupuytren's disease. Analyzing the biobanks separately, we find 37 (UK Biobank), 19 (FinnGen), and 2 (MGI) haplotypes with genome-wide significance. To increase the statistical power, we performed a meta-analysis of the 3 biobanks based on single nucleotide variants present in all biobanks (*N* = 12,940,999 variants). To control for false positives, we calculated the genomic inflation factor which was found to be reasonable (λ = 1.10; supplementary fig. S1, Supplementary Material online). In total, we find 61 genetic loci of genome-wide significance (fig. 1; supplementary table S1, Supplementary Material online), out of which 24 are not significant in any biobank singly. The meta-analysis identifies 21 novel risk loci in addition to the ones previously identified by 2 GWASs (Dolmans et al. 2011, Ng et al. 2017).

### Neandertal Variants Increase the Risk for Dupuytren's Disease

First, we investigate the prevalence of Dupuytren's disease in individuals of European and African descent. In the MGI biobank, we find a Dupuytren's disease prevalence of 0.49% (95% CI: 0.26–0.83%) among individuals of primarily African ancestry, whereas the corresponding prevalence among individuals of primarily European ancestry is 1.37% (95% CI: 1.25–1.48%; *t*-test, 2-tailed, *P* = 0.00012). Similarly, a lower prevalence of Dupuytren's disease has been reported in the Million Veteran Program (Saboeiro et al. 2000) among individuals of primarily African ancestry (0.13%, 95% CI: 0.12–0.14%) compared with individuals of primarily European descent (0.73%, 95% CI: 0.72–0.75%). Taken together, European ancestry is associated with a higher risk for Dupuytren's disease than African ancestry (supplementary fig. S2, Supplementary Material online).

Given the rarity of Dupuytren's contracture among individuals of primarily African ancestry, we analyzed if any of the 61 risk loci harbor alleles inherited from Neandertals. We devised a novel algorithm, building on a recently described method (Chen et al. 2020), to identify archaic tracts in modern humans (see Materials and Methods). In brief, we identified haplotypes similar to the genomes of Neandertals and Denisovans (collectively referred to as “archaic humans”), now extinct human forms that lived in Western and Eastern Eurasia, respectively, until at least approximately ∼42,000 years ago (Devièse et al. 2021). We investigated whether such haplotypes overlapped with the most significant variant in each locus associated with Dupuytren's contracture and whether the most significant variant cosegregated with archaic haplotypes. Present-day genomes contain haplotypes that are similar to archaic genomes both because they inherited haplotypes from populations ancestral to both modern humans and archaic humans and because modern humans interbred with archaic groups when they met less than 100,000 years ago (Sankararaman et al. 2012). In order to restrict the analyses to recently introgressed archaic variants, we only considered haplotypes longer than 0.023 cM, which corresponds to a significance threshold of *P* < 0.05 for being derived from the ancestral population (adjusted for multiple comparisons; see Materials and Methods).

**Fig. 2.**
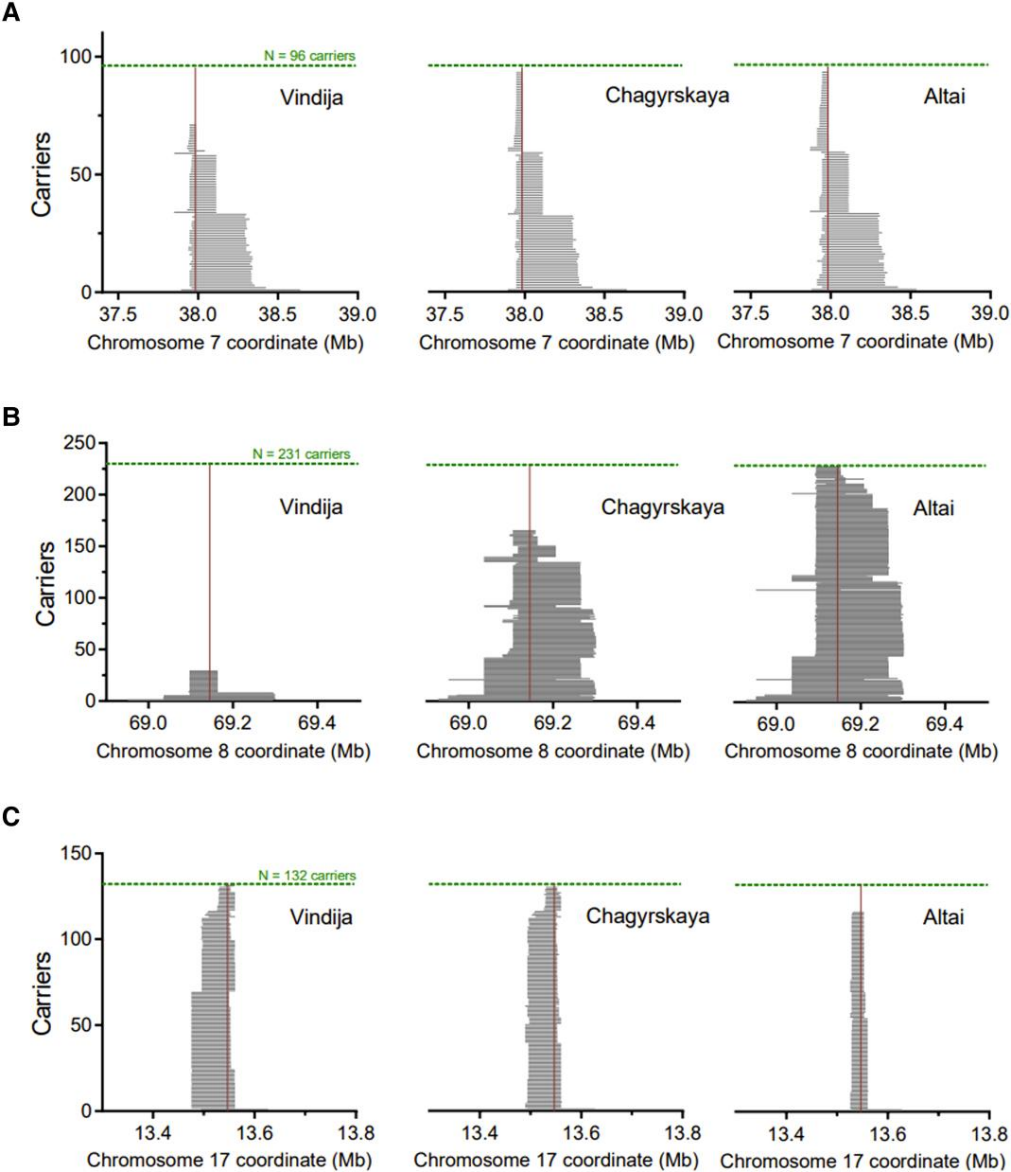
Archaic haplotypes in present-day carriers of 3 Dupuytren's disease risk variants. Three Neandertal genomes (Vindija, Chagyrskaya, and Altai) and 1 Denisova genome were used as archaic references (Meyer et al. 2012, Prüfer et al. 2014, 2017, Mafessoni et al. 2020). Archaic haplotype lengths were calculated using identity by descent (Chen et al. 2020). (*A*–*C*) Haplotype lengths and counts associated with (*A*) the second major risk allele (chr7:37,984,972*G*/A, *hg38*), (*B*) the third major risk allele (chr8:69,142,848*G*/A, *hg38*), and (*C*) the chr17 risk allele (chr17:13,547,115*C*/T, *hg38*). See table 1 for risk associations and haplotype counts.

None of the loci harbored alleles that cosegregated (*r*^2^ > 0.2) with Denisovan haplotypes (supplementary table S2, Supplementary Material online). In contrast, we identified 3 loci in which the variant most significantly associated with Dupuytren's disease cosegregates with Neandertal haplotypes (*r*^2^ > 0.95, table 1). These loci include the second (rs17171240, chr7:37,984,972G/A, *hg38*, *P* = 6.4 × 10^−132^) and third (rs652483, chr8:69,142,848G/A, *hg38*, p = 9.2 × 10^−69^) most significant variants. In addition to these major risk factors, a third variant is also inherited from Neandertals (rs34017855, chr17:13,547,115C/T, *hg38*, p = 1.1 × 10^−8^). For these 3 variants, the most significant variant cosegregates with Neandertal haplotypes irrespectively of whether we use the “Altai” (Prüfer et al. 2014), the “Vindija” (Prüfer et al. 2017), or the “Chagyrskaya” (Mafessoni et al. 2020) Neandertal genomes. We find that for the variants on chromosome 7 and 8, using the “Altai” or “Chagyrskaya” Neandertal genomes as the introgressing Neandertal, result in a cosegregation of *r*^2^ > 0.95, but *r*^2^ = 0.72 and *r*^2^ = 0.35, respectively, using the “Vindija” genome (fig. 2; table 1). Thus, these 2 variants harbor Neandertal haplotypes more similar to the “Altai” and “Chagyrskaya” Neandertal genomes. For the variant on chromosome 17, we find a stronger cosegregation to the “Vindija” and “Chagyrskaya” Neandertal (*r*^2^ > 0.95) than for the “Altai” Neandertal genome (*r*^2^ = 0.84; table 1). Using the Denisovan genome (Meyer et al. 2012) as the introgressing archaic genome, we observe low correlations for all 61 variants influencing the risk for Dupuytren's disease (*r*^2^ < 0.2). However, we caution that IBDmix is not optimized for identifying haplotypes of Denisova origin (Chen et al. 2020). Since the choice of recombination map can influence the conclusion whether a haplotype is the result of gene flow or not, we substituted the African American-based recombination map (Zhou et al. 2020) for an Icelandic high-resolution recombination map (Halldorsson et al. 2019), or for a map inferred from identity-by-descent among European Americans (Zhou et al. 2020). We find Neandertal introgression in all 3 loci irrespective of recombination map used (supplementary tables S3 and S4, Supplementary Material online; see Materials and Methods).

**Table 1.** Neandertal-introgressed Risk Variants Associated With Dupuytren's Disease.

Chr	Position	Archaic haplotype LD *r*^2^(number)				Carriers 1000G (allele)	rsID	Reference allele	Alternative allele	OR (95% CI)
		Vindija	Chagyrskaya	Altai	Denisova					
7	37,984,972	0.72 (71)	0.95 (93)	0.95 (93)	0.029 (4)	96 (A)	rs17171240	G	A	1.79 (1.71–1.88)
8	69,142,848	0.35 (29)	0.73 (166)	0.99 (229)	0.000 (0)	231 (G)	rs652483	G	A	0.72 (0.70–0.75)
17	13,547,115	0.97 (131)	0.96 (130)	0.84 (117)	0.001 (11)	132 (T)	rs34017855	C	T	1.14 (1.09–1.20)

Archaic-like haplotypes cosegregating with risk variants (*r*^2^ > 0.5 among Europeans) with a length of > 0.023 cM (see Materials and Methods). Odds ratios (OR) for association with Dupuytren's disease are derived from the meta-analysis of UK Biobank, FinnGen, and MGI. Coordinates shown in *hg38*.

**Table 2.** Credible Set of Causal Variants in the Neandertal Haplotype.

Chr	Position	Ref	Alt	Anc.	Archaic	rsID	OR	*P* value	PIP	CADD	Reg. feature
7	37,984,972	G	A	G	G/A	rs17171240	1.79	6.4 × 10^−132^	0.21	7.3	Open chromatin
7	37,984,802	G	A	A	G/A	rs2044830	1.79	7.4 × 10^−132^	0.18	10.0	Open chromatin
7	37,984,011	T	C	T	T/C	rs117329120	1.79	8.2 × 10^−132^	0.16	1.1	No overlap
7	37,983,798	A	G	A	A/G	rs117575966	1.79	8.7 × 10^−132^	0.15	2.2	No overlap
7	37,964,710	G	A	G	G/A	rs117387543	1.78	2.0 × 10^−131^	0.07	1.5	No overlap
7	37,964,701	A	G	A	A/G	rs114929416	1.78	2.0 × 10^−131^	0.06	1.7	No overlap
7	37,964,907	A	G	A	A/G	rs79590116	1.78	2.0 × 10^−131^	0.06	4.5	No overlap
7	37,966,121	T	A	T	T/A	rs75182114	1.77	2.4 × 10^−131^	0.06	0.1	No overlap
7	37,976,920	T	G	T	T/G	rs74335252	1.79	2.5 × 10^−131^	0.05	2.1	No overlap

95% credible set derived from fine-mapping assuming one causal variant (Locuszoom; Pruim et al. 2010, Boughton et al. 2021). Archaic refers to alleles present in the Vindija, Chagyrskaya, and Altai Neandertal and Denisova genomes (Meyer et al. 2012, Prüfer et al. 2014, 2017, Mafessoni et al. 2020). Regulatory features are derived from Ensembl (Zerbino et al. 2015). Coordinates are in *hg38.*

Ref., reference allele; Alt., alternative allele; Anc., ancestral allele; OR, odds ratio; PIP, posterior inclusion probability based on fine-mapping; CADD, Combined Annotation Dependent Depletion score (Rentzsch et al. 2019).

Next, we evaluate if these Neandertal-derived haplotypes are associated with an increased risk, or protection against Dupuytren's disease. We find that all 3 Neandertal haplotypes are associated with an increased risk for Dupuytren's disease (table 1). Note, however, that for the chromosome 8 locus the G allele tagging the introgressed haplotypes in this locus is not observed in the sequenced Neandertal genomes. This variant is likely to represent an haplotype not yet sequenced from Neandertals. Another less likely possibility is that it is the result of a mutation early after introgression. Assuming a simple additive model, the combined odds ratio of carrying all 3 Neandertal risk alleles is 2.83 (95% CI = 2.62–3.05).

In a previous genome-wide scan (Racimo et al. 2017) the Neandertal haplotype associated with Dupuytren's disease on chromosome 8 has been identified as positively selected. It remains to be elucidated, however, what putatively positively selected trait this haplotypes might influence. In FinnGen and UK Biobank, this polymorphism is not associated with any trait other than Dupuytren's disease.

A rough calculation suggests that 8.4% of the heritability (h^2^) explained by the 61 genome-wide hits in our meta-analysis can be attributed to the 3 genetic risk factors of Neandertal origin (see Materials and Methods). In line with this, we find that the difference in polygenic risk score between cases and controls for Dupuytren's disease (see Materials and Methods) is reduced if the 3 loci harboring Neandertal haplotypes are removed from the analysis (supplementary fig. S3, Supplementary Material online).

### Functional Features of the Major Neandertal Risk Factor

**Fig. 3.**
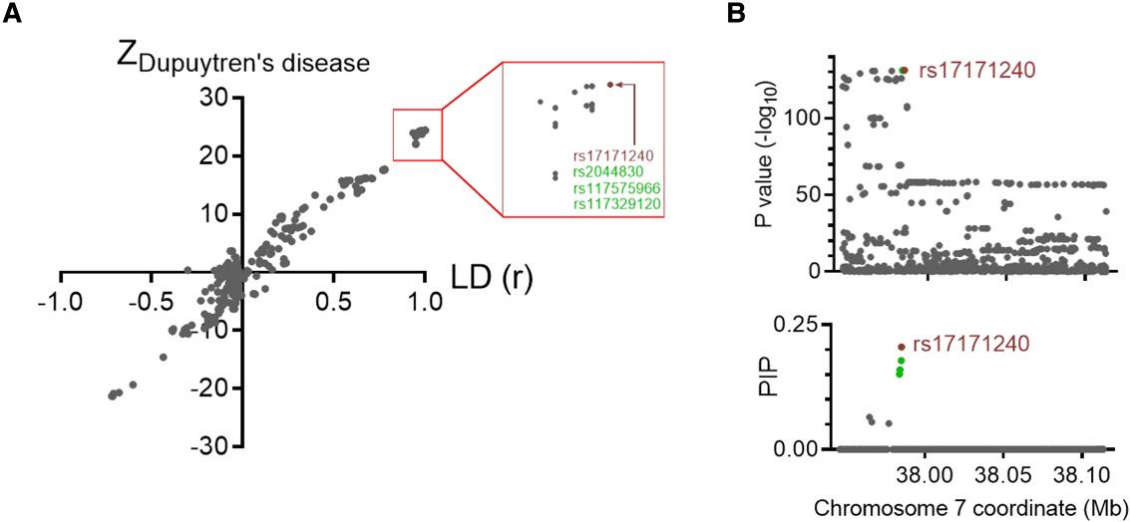
Fine-mapping of the Neandertal haplotype. (*A*) Z-score for association with Dupuytren's disease (Z_Dupuytren's disease_) as a function of linkage disequilibrium (*r*) with the most significant risk variant on chromosome 7 (rs17171240, chr7:37,984,972*G*/A, *hg38*; marked in red). Variants marked in green are in full linkage disequilibrium (*r*^2^ = 1.00) with rs17171240. Linkage disequilibrium data are from the 1000 Genomes Project (Auton et al. 2015). (*B*) *P* values for Dupuytren's disease associations (upper panel) and posterior inclusion probabilities (PIP; lower panel) for causal variants derived from fine-mapping under the assumption of one causal variant (Pruim et al. 2010, Boughton et al. 2021).

In an attempt to identify putative causal genetic variants of the strongest risk factor inherited from Neandertals (fig. 2), we perform a fine-mapping analysis of the risk locus. Plotting the variant association strengths, corrected for error sizes (i.e., Z-scores), for Dupuytren's disease against linkage disequilibrium with the strongest associated variant in the locus, we observe a straight line (fig. 3*A*). This suggests that the weakly associated variants gain their association with the phenotype by cosegregating with more strongly associated variants. Thus, the assumption of one causal variant is parsimonious. Based on this assumption, a fine-mapping analysis identifies 9 single-nucleotide polymorphisms (SNPs) in the 95% credible set (fig. 3*B*; table 2; see Materials and Methods), of which the 2 most significant variants are most impactful based on *in silico* effect prediction (Rentzsch et al. 2019; Combined Annotation Dependent Depletion scores of 7.3 and 10.0, respectively). We note that both of these variants fall within an open chromatin region in dermal fibroblasts (chr7:37,984,714–37,985,157, *hg38*; ENSR00000211077, Zerbino et al. 2015), whereas the other 7 variants do not. Of these 2 putative causal SNPs, the risk allele is ancestral for one of the SNPs (rs17171240) and for the other SNP it is derived among Neandertals and Denisovans (rs2044830, chr7:37,984,802G/A, *hg38*). These 2 SNPs are in perfect linkage disequilibrium among Europeans in the 1000 Genomes Project (Auton et al. 2015).

**Fig. 4.**
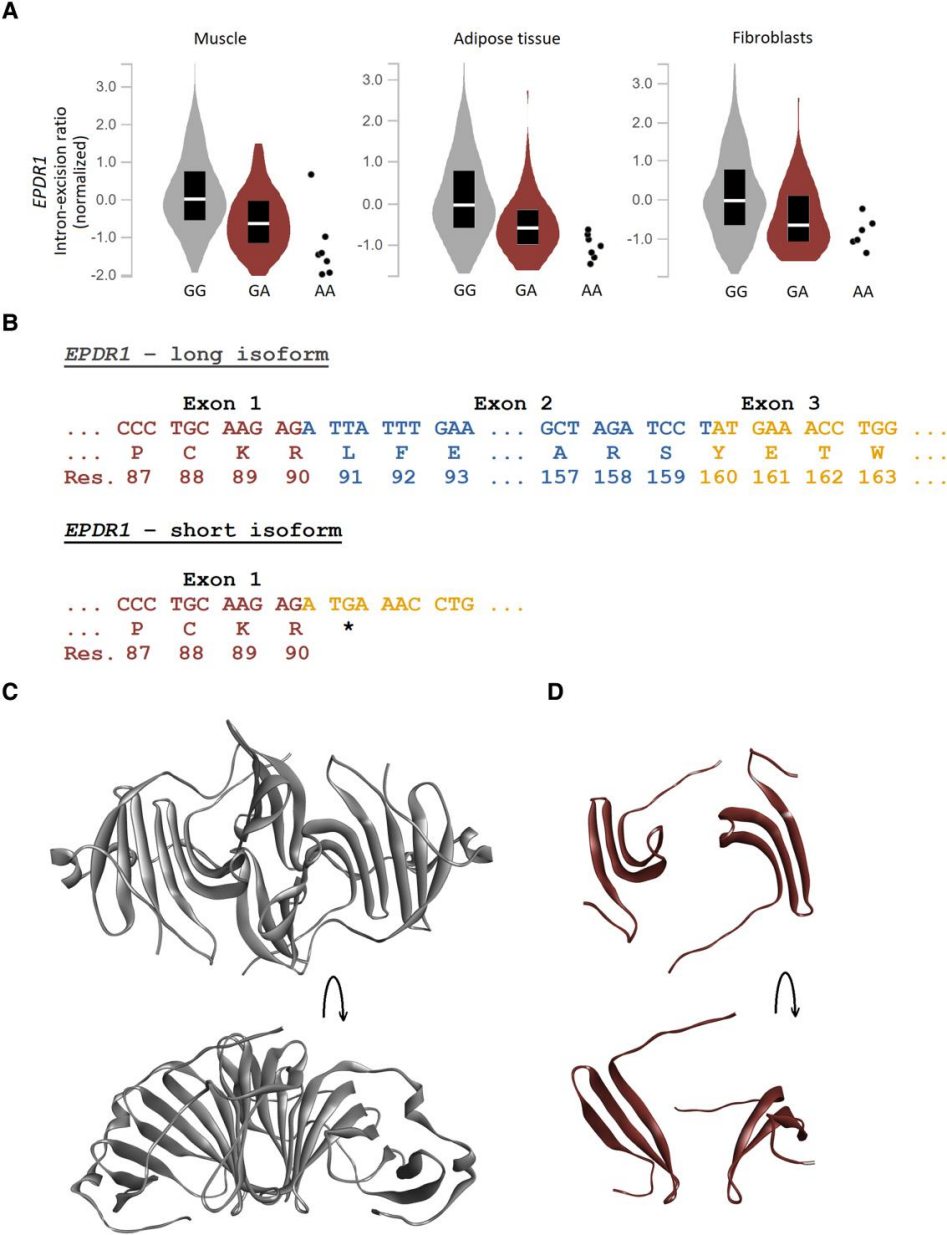
The Neandertal haplotype is associated with alternative splicing of *EPDR1*. (*A*) Genotype-tissue expression project-derived *cis-*splicing quantitative trait loci for the Neandertal haplotype lead variant (Lonsdale et al. 2013). rs17171240 (chr7:37,984,972*G*/A, *hg38)* reduces the intron excision ratio of the *EPDR1* transcript in skeletal muscle (normalized effect size [NES] = −0.77, *P* = 1.7 × 10^−23^, GG, *n* = 581, GA, *n* = 118, AA = 7; left panel), adipose tissue (NES = −0.63, *P* = 1.1 × 10^−11^, GG, *n* = 481, GA, *n* = 93, AA = 7; middle panel), and cultured fibroblasts (NES = −0.60, *P* = 5.9 × 10^−8^, GG, *n* = 393, GA, *n* = 84, AA = 6; right panel). (*B*) The long *EPDR1* isoform (*NM_017549.5*) consists of exons 1–3, translating into the 290 amino acid ependymin related protein 1 (EPDR1). The short *EPDR1* isoform (*NM_001242946.2*) is produced by alternative splicing at base pair 570, creating an out-of-frame stop codon (TGA) that translates into a truncated protein of 90 amino acids. (*C*) X-ray crystallography-derived structure of EPDR1 (PDB: 6E7*O*; Wei et al. 2019) in dimeric form from 2 angles. Amino acids 37/38 to 222, respectively, are shown. (*D*) Structure of the truncated EPDR1 (amino acids 37/38 to 90) from 2 angles. The lipid binding pocket and dimerization interface are disrupted.

None of the alleles in the 95% credible set fall within an exon. We therefore explored possible effects on mRNA splicing and expression using the genotype-tissue expression project (GTEx; Lonsdale et al. 2013). We find that the strongest risk factor inherited from Neandertals is associated with altered splicing of *EPDR1* transcripts in 3 tissues relevant for Dupuytren's disease; muscle (*P* = 1.7 × 10^−23^), adipose tissue (*P* = 1.1 × 10^−11^), and cultured fibroblasts (*P* = 5.9 × 10^−8^; fig. 4*A*).

For the canonical transcript of *EPDR1* (*NM_017549.5*), the region chr7:37,948,840–37,949,048 (*hg38*) encodes the second exon. In the GTEx data, we find that the strongest risk factor inherited from Neandertals is associated with the region chr7:37,921,208–37,950,200 (*hg38*) being intronic (GTEx splicing data identifies differential intron excision and not exon expression; Li et al. 2018). Thus, this allele is associated with expression of a transcript (*NM_001242946.2*), lacking the second exon of the canonical transcript, predicted to result in a frameshift truncation after the 90th amino acid residue of the translated protein (fig. 4*B*). The full-length canonical transcript encodes the 290 amino acids long, homodimer-forming, lipid-binding, Ependymin Related Protein 1 (EPDR1; Wei et al. 2019; fig. 4*C*). The truncated protein encoded by the alternative transcript lacks the dimerization interface and large parts of the lipid-binding pocket (fig. 4*D*).

**Fig. 5.**
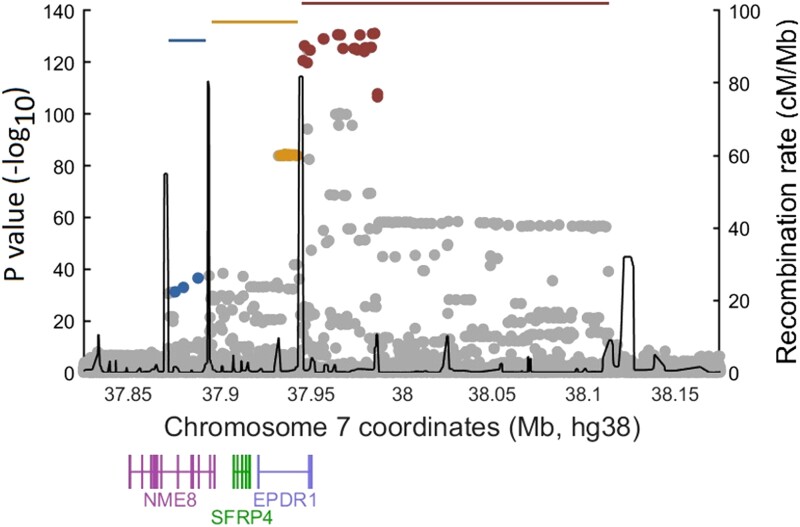
Genetic architecture of the chromosome 7 risk region. Association strengths with Dupuytren's disease for the risk region located on chromosome 7. Notably, there are 3 independent haplotypes (blue, yellow, and red horizontal lines) associated with Dupuytren's disease, separated by recombination hotspots. In each window, the variants in high LD (*r*^2^ > 0.8) are colored in red (rs17171240, chr7:37,984,972*G*/A, *hg38*; index for Neandertal haplotype), yellow (rs2598104, chr7:37,937,647 T/C, *hg38*), and blue (rs6462793, chr7:37,887,948*G*/C, *hg38*), respectively. Recombination rates are derived from the 1000 Genomes Project (Auton et al. 2015).

Upstream of the Neandertal haplotype, recombination hotspots delineate 2 separate regions (fig. 5) associated with Dupuytren's disease, chr7:37,933,225-37,942,359 (tagged by rs2598104, chr7:37,937,647 T/C, *hg38*) and chr7:37,871,701-37,887,948 (tagged by rs6462793, chr7:37,887,948G/C, *hg38*). These genetic variants do not cosegregate with the Neandertal haplotype (*r*^2^ < 0.1) and are thus independent genetic risk factors for Dupuytren's disease. Expression data from blood (Võsa et al. 2021) show that both genomic regions are associated with decreased *EPDR1* expression (rs2598104, *P* = 3.3 × 10^−310^; rs6462793, *P* = 1.9 × 10^−98^; see supplementary table S5, Supplementary Material online). In contrast to the Neandertal haplotype, there is no indication that rs2598104 or rs6462793 affect splicing of *EPDR1* in GTEx (Lonsdale et al. 2013).

## Discussion

There are geographical differences in the amount and type of archaic ancestry. People from Africa south of Sahara have little, or minimal, ancestry from Neandertals or Denisovans, whereas people with roots outside of Africa have inherited 1–2% of their genome from Neandertals (Chen et al. 2020). Denisovan ancestry is found in East Asian and Oceanian populations, where some populations in Oceania have up to ∼5% of Denisovan ancestry (Larena et al. 2021). Given these regional differences, it is likely that archaic gene variants contribute to some phenotypes that are primarily seen only in certain populations. Here, we focus on one such regional phenotype, Dupuytren's disease.

The present meta-analysis identifies 61 independent genetic risk variants associated with Dupuytren's disease. We find that the second most important genetic risk factor located on chromosome 7, which confers an odds ratio of ∼1.8 (*P* = 6.4 × 10^−132^; fig. 3), is of Neandertal origin. By analyzing mRNA data from relevant tissues (muscle, adipose tissue, and fibroblasts), we find that the genetic risk factor is associated with a splicing variant of *EPDR1* resulting in an inferred truncation of the protein. Previous work has suggested the gene *SFRP4* as the causal agent in this locus (Ng et al. 2017, Jin et al. 2022), although *EPDR1* has also been implicated in Dupuytren's disease susceptibility (Staats et al. 2016). The present data suggest *EPDR1* as a causal gene. EPDR1 is a lysosomal protein and recent structural determination using X-ray crystallography suggests that the functional protein complex is a dimer and contains a lipid binding site (Wei et al. 2019), which are disrupted by the alternative splicing caused by the Neandertal haplotype. Although the exact pathophysiological mechanism for how the truncation of EPDR1 might contribute to the development of Dupuytren's disease deserves further investigation, it is worth noting that this protein is suggested to be involved in myofibroblast contractility (Staats et al. 2016).

Fine-mapping of the Neandertal haplotype suggests 2 equally likely causal variants located in an open chromatin region separated by only 170 base pairs (rs17171240, chr7:37,984,972G/A and rs2044830, chr7:37,984,802G/A, *hg38*). These variants cosegregate in modern humans and the Neandertal state of them are associated with an increase in risk. Given that one of the SNPs harbors an ancestral risk allele and the other a Neandertal-derived risk allele, we can only speculate as to whether the risk for Dupuytren's disease for this locus partly represents an ancestral state or a phenotype that emerged among Neandertals.

In addition to the risk locus on chromosome 7 (rs17171240), which is the second-most strongly associated variant in our meta-analysis, we find 2 additional loci where the risk allele is inherited from Neandertals, on chromosomes 8 (rs652483) and 17 (rs34017855), respectively. The chromosome 8 variant is the third most strongly associated variant in the dataset, whereas the chromosome 17 variant is the 52nd variant in terms of association strength.

Assuming that Neandertal and modern human haplotypes have equal effects and frequencies, the expected Neandertal heritability to a genetic trait is reflected by the average Neandertal ancestry among Europeans (∼2%). Under such a scenario, Dupuytren's disease is approximately 4 times more influenced by Neandertal gene flow than expected (8.4% vs. ∼2%). However, Neandertal haplotypes typically have substantially lower frequencies than averages modern human haplotypes. The average minor allele frequency in the GWAS catalogue is 26.6% (Auton et al. 2015), similar to the 24.6% minor allele frequency for the loci not harboring Neandertal haplotypes in our meta-analysis. Assuming that half of the genome contains gene flow from Neandertals, Neandertal haplotypes have an average frequency of ∼4%. Using this allele frequency for the loci harboring Neandertal haplotypes and the average catalogue frequency for the rest of loci, we find that the expected heritability explained by Neandertal admixture is ∼0.4% (see Materials and Methods). Under these assumptions, the observed influence of Neandertal ancestry on Dupuytren's disease is ∼20-fold larger than expected. Taken together, these calculations suggest that admixture with Neandertals has a substantial impact on the prevalence of Dupuytren's disease in Europe.

## Materials and Methods

### Study Population

Summary statistics for Dupuytren's disease (International Classification of Diseases, Ninth Revision, Clinical Modification; code 728.71) were retrieved from the UK Biobank (TOPMED-imputed) (https://pan.ukbb.broadinstitute.org/), FinnGen (release 7; https://r7.finngen.fi), and the MGI (https://pheweb.org/, freeze 3). The 3 biobanks mainly include individuals of European descent.

### Meta-analysis

For the meta-analysis, we intersected SNPs present in the UK Biobank (*N* = 56,936,751), FinnGen (*N* = 16,383,194), and MGI (*N* = 39,981,535), resulting in 12,940,999 unique variants present in all 3 biobanks. We meta-analyzed the effects in each of the studies using the inverse-variance weighted method and fixed-effects (Balduzzi et al. 2019). To avoid floating point errors for *P* < 10^−300^, numerical approximation was performed based on Z-values using NIntegrate in Mathematica 12 (Wolfram Research, Inc., Champaign, IL). Loci with genome-wide significant (*P* < 5 × 10^−8^) association with Dupuytren's disease were identified using LocusZoom (Pruim et al. 2010, Boughton et al. 2021).

### Gauging Neandertal Ancestry in the Genetic Predisposition to Dupuytren's Disease

Given that only about half of any archaic genome is mapped, we did not require the most significant variant in each locus to be present in an archaic genome and missing in some modern human reference panel with minimal archaic ancestry, as we have done previously (Zeberg et al. 2020a, 2020b). Instead, we investigated to what extent the most significant polymorphism in each locus cosegregated with overlapping archaic tracts. We used the software IBDmix (Chen et al. 2020) to identify archaic tracts, using 1 of the 4 high-coverage archaic genomes available (Meyer et al. 2012, Prüfer et al. 2014, 2017, Mafessoni et al. 2020) as an introgressing genome and 503 European genomes included in the 1000 Genomes Project (Auton et al. 2015). IBDmix was run with default parameters, except for a more conservative LOD-score threshold of 4, as has been suggested (Chen et al. 2020). To determine if archaic tracts were homozygously present, we used bcftools v1.14 to call runs of homozygosity. PLINK v1.9 was used to determine the linkage disequilibrium (unphased *r*^2^) between a given variant and overlapping archaic haplotypes. To exclude that an archaic-like sequence originates from the common ancestor, we calculated the minimal genetic length that would exclude incomplete lineage sorting. We used a mathematical framework based on previous work (Huerta-Sánchez et al. 2014), but in contrast to performing the calculations on the physical length and using years, we directly used genetic length and branch lengths in generations, as follows. Let *L* be the expected genetic length of a shared ancestral sequence given by the inverse of the total branch length. The number of generations since the common ancestor of Neandertals and modern humans has been estimated to ∼21,500 (Sjödin et al. 2021) and the archaic admixture took place ∼2,000 generations ago (Sankararaman et al. 2012). Thus, the total branch length for a recent gene-flow model is 2 × 21,500–2,000 = 41,000 generations, and therefore the expected genetic length of a shared sequence is *L* = 1/(41,000 generations) = 1/410 cM. Conditioning on observing the archaic like tract on both branches, the probability of a length of at least *m* follows a Gamma distribution with shape parameter 2, and rate parameter = 1/*L*. We required Bonferroni correction for the number of loci tested, giving a significance threshold of *P* = 0.05/61. Finally, we solved numerically the equation 1—GammaCDF(*m*, shape = 2, rate = 410) = 0.05/61, where GammaCDF is the cumulative gamma distribution, yielding *m* = 0.023 cM. Hence, only tracts longer than 0.023 cM were considered significant for gene flow.

A limitation with using genetic distances is that the historical recombination map is largely unknown, particularly for archaic populations. A recombination map estimated from African populations, however, may be more accurate given that the vast majority of the modern human branch predates the major out-of-Africa exodus. Moreover, out-of-Africa populations differ in recombination rates due to drift that occurred in the out-of-Africa bottleneck (e.g., due to alleles in *PRDM9*; Paigen and Petkov 2018). We decided to use a recent map based on 2,046 unrelated individuals of African ancestry from the Jackson Heart Study (Zhou et al. 2020). For control purposes, we iterated the analysis using recombination maps from the Framingham Heart Study (Americans; Zhou et al. 2020) and deCODE (Icelandic; Halldorsson et al. 2019). At the end of the chromosomes where genetic distances were missing, we assumed a recombination rate of 1 cM/Mb (i.e., a cutoff of 23 kb).

### Fine-mapping Analysis

We performed a Bayesian fine-mapping using LocusZoom (Pruim et al. 2010, Boughton et al. 2021). For this analysis, we restricted the haplotype-based recombination hotspots derived from the 1000 Genomes Project (*hg19*) which were lifted over to *hg38* coordinates using the University of California, Santa Cruz, Genome Browser (https://genome.ucsc.edu/). To investigate the ancestral state among the putative causal SNPs, we obtained the ancestral alleles from Ensembl (Cunningham et al. 2022).

### Linkage Disequilibrium

Linkage disequilibrium statistics were derived from the 1000 Genomes Project (Auton et al. 2015) using Tomahawk (https://mklarqvist.github.io/tomahawk/). European populations (Finnish in Finland, British in England and Scotland, Iberian in Spain, Toscani in Italia, and Utah Residents with Northern and Western European Ancestry) were used as references to reflect the included biobank participants.

### Quantitative Trait Loci Analyses

Alterations in transcript expression levels for single-nucleotide variants were determined from *cis*-expression quantitative trait loci (eQTLs) data, retrieved from the Genotype-Tissue Expression Project version 8 (https://gtexportal.org/; Lonsdale et al. 2013). Correspondingly, *cis*-splicing quantitative trait loci (sQTLs) data were derived from GTEx. sQTL data are reported as transcript intron-excision ratios, which were calculated using LeafCutter (Li et al. 2018). Blood *cis*-eQTLs were derived from the eQTLGen Consortium (Võsa et al. 2021).

### Heritability Calculations and Polygenic Risk Scoring

When estimating heritability for genetic variants for case-control studies, a linear transformation exists such that *h^2^ ≈ k × f × (1−f) × β*, where *β* is the effect estimate, *f* the allele frequency and *k* a scaling factor dependent on the population, sample prevalence and the liability threshold (Lee et al. 2011). We calculated the sum of *h^2^* for the most significant variant in all 61 genome-wide loci (*h^2^_total_*) and the sum for the 3 variants inherited from Neandertals, *h^2^_Neandertals_*. Dividing *h^2^_Neandertals_* with *h^2^_total_* (thereby canceling *k*) results in that 8.4% of the heritability explained by the 61 genome variants could be attributed to Neandertal gene flow. For the expected Neandertal contribution to any genetic trait, we used the same formulae as above assuming 2% of the loci harbor Neandertal haplotypes and using various assumptions regarding the allele frequencies (see Discussion).

Polygenic risk score was calculated per group or individual as a sum of effect sizes per risk allele multiplied by the number of risk alleles. First, we calculated the polygenic risk score for all loci associated with Dupuytren's disease by using allele frequency data for risk alleles based on cases and controls in the FinnGen biobank. Next, we calculated polygenic risk scores per population in the 1000 Genomes Project (Auton et al. 2015) based on African, European, East Asian, and South Asian populations. The polygenic risk score probability distribution was determined for all 61 associated risk variants and compared with the corresponding distribution in the absence of the 3 Neandertal-derived risk loci.

### Biobank Ethics Statements and Biobank Materials and Methods (FinnGen and MGI)

Patients and control subjects in FinnGen provided informed consent for biobank research, based on the Finnish Biobank Act. Alternatively, separate research cohorts, collected prior the Finnish Biobank Act came into effect (in September 2013) and start of FinnGen (August 2017), were collected based on study-specific consents and later transferred to the Finnish biobanks after approval by Fimea (Finnish Medicines Agency), the National Supervisory Authority for Welfare and Health. Recruitment protocols followed the biobank protocols approved by Fimea. The Coordinating Ethics Committee of the Hospital District of Helsinki and Uusimaa (HUS) statement number for the FinnGen study is Nr HUS/990/2017.

The FinnGen study is approved by Finnish Institute for Health and Welfare (permit numbers: THL/2031/6.02.00/2017, THL/1101/5.05.00/2017, THL/341/6.02.00/2018, THL/2222/6.02.00/2018, THL/283/6.02.00/2019, THL/1721/5.05.00/2019, THL/1524/5.05.00/2020, and THL/2364/14.02/2020), Digital and population data service agency (permit numbers: VRK43431/2017-3, VRK/6909/2018-3, VRK/4415/2019-3), the Social Insurance Institution (permit numbers: KELA 58/522/2017, KELA 131/522/2018, KELA 70/522/2019, KELA 98/522/2019, KELA 138/522/2019, KELA 2/522/2020, KELA 16/522/2020, Findata THL/2364/14.02/2020), and Statistics Finland (permit numbers: TK-53-1041-17 and TK/143/07.03.00/2020 (earlier TK-53-90-20).

The Biobank Access Decisions for FinnGen samples and data utilized in FinnGen Data Freeze 7 include the following: THL Biobank BB2017_55, BB2017_111, BB2018_19, BB_2018_34, BB_2018_67, BB2018_71, BB2019_7, BB2019_8, BB2019_26, BB2020_1, Finnish Red Cross Blood Service Biobank 7.12.2017, Helsinki Biobank HUS/359/2017, Auria Biobank AB17-5154 and amendment #1 (August 17 2020), Biobank Borealis of Northern Finland_2017_1013, Biobank of Eastern Finland 1186/2018 and amendment 22 §/2020, Finnish Clinical Biobank Tampere MH0004 and amendments (21.02.2020 & 06.10.2020), Central Finland Biobank 1-2017, and Terveystalo Biobank STB 2018001.

### MGI Cohort

The MGI comprises genome-wide data and electronic health information for patients recruited via Michigan Medicine (Zawistowski et al. 2023). Informed written consent was obtained from each participant, who donated a blood sample for genetic analysis and underwent a comprehensive medical assessment. Data were collected according to Declaration of Helsinki principles. Written consent forms and study protocols were reviewed and approved by the University of Michigan Medical School Institutional Review Board.

## Supplementary Material

msad130_Supplementary_Data

## Data Availability

Data generated or analyzed in this study are included in this article and its Supplementary Information Files.

## References

[msad130-B1] Auton A , Abecasis GR, Altshuler DM, Durbin RM, Abecasis GR, Bentley DR, Chakravarti A, Clark AG, Donnelly P, Eichler EE, et al 2015. A global reference for human genetic variation. Nature 526:68–74. 26432245 10.1038/nature15393 PMC4750478

[msad130-B2] Balduzzi S , Rücker G, Schwarzer G. 2019. How to perform a meta-analysis with R: a practical tutorial. Evid Based Ment Health 22:153–160. 31563865 10.1136/ebmental-2019-300117 PMC10231495

[msad130-B3] Beighton P , Valkenburg HA. 1974. Bone and joint disorders on Tristan da Cunha. S Afr Med J. 48:743–747. 4274709

[msad130-B4] Boughton AP , Welch RP, Flickinger M, VandeHaar P, Taliun D, Abecasis GR, Boehnke M. 2021. Locuszoom.js: interactive and embeddable visualization of genetic association study results. Bioinformatics 37:3017–3018 . 33734315 10.1093/bioinformatics/btab186 PMC8479674

[msad130-B5] Burge P . 1999. Genetics of Dupuytren's disease. Hand Clin. 15:63–71. 10050243

[msad130-B6] Chen L , Wolf AB, Fu W, Li L, Akey JM. 2020. Identifying and interpreting apparent Neanderthal ancestry in African individuals. Cell 180:677–687.e16. 32004458 10.1016/j.cell.2020.01.012 PMC12805117

[msad130-B7] Cunningham F , Allen JE, Allen J, Alvarez-Jarreta J, Amode MR, Armean IM, Austine-Orimoloye O, Azov AG, Barnes I, Bennett R, et al 2022. Ensembl 2022. Nucleic Acids Res. 50:D988–D995. 34791404 10.1093/nar/gkab1049 PMC8728283

[msad130-B8] Devièse T , Abrams G, Hajdinjak M, Pirson S, De Groote I, Di Modica K, Toussaint M, Fischer V, Comeskey D, Spindler L, et al 2021. Reevaluating the timing of Neanderthal disappearance in northwest Europe. Proc Natl Acad Sci U S A. 118:e2022466118. 10.1073/pnas.2022466118 PMC7999949 33798098

[msad130-B9] Dolmans GH , Werker PM, Hennies HC, Furniss D, Festen EA, Franke L, Becker K, van der Vlies P, Wolffenbuttel BH, Tinschert S, et al 2011. Wnt signaling and Dupuytren's disease. N Engl J Med. 365:307–317. 21732829 10.1056/NEJMoa1101029

[msad130-B10] Dutta A , Jayasinghe G, Deore S, Wahed K, Bhan K, Bakti N, Singh B. 2020. Dupuytren's contracture – current concepts. J Clin Orthop Trauma. 11:590–596. 32684695 10.1016/j.jcot.2020.03.026 PMC7355095

[msad130-B11] Halldorsson BV , Palsson G, Stefansson OA, Jonsson H, Hardarson MT, Eggertsson HP, Gunnarsson B, Oddsson A, Halldorsson GH, Zink F, et al 2019. Characterizing mutagenic effects of recombination through a sequence-level genetic map. Science 363:eaau1043. 30679340 10.1126/science.aau1043

[msad130-B12] Huerta-Sánchez E , Jin X, Asan, Bianba Z, Peter BM, Vinckenbosch N, Liang Y, Yi X, He M, Somel M, et al 2014. Altitude adaptation in Tibetans caused by introgression of denisovan-like DNA. Nature 512:194–197. 25043035 10.1038/nature13408 PMC4134395

[msad130-B13] Jin R , Zhu W, Xu J, Gu J, Deng A. 2022. Effect of nanoparticle-mediated delivery of SFRP4 siRNA for treating Dupuytren disease. Gene Ther. 30:31–40. 35347304 10.1038/s41434-022-00330-9

[msad130-B14] Larena M , McKenna J, Sanchez-Quinto F, Bernhardsson C, Ebeo C, Reyes R, Casel O, Huang JY, Hagada KP, Guilay D, et al 2021. Philippine Ayta possess the highest level of Denisovan ancestry in the world. Curr Biol. 31:4219–4230.e10. 34388371 10.1016/j.cub.2021.07.022 PMC8596304

[msad130-B15] Larsen S. , Krogsgaard D. G., Larsen LA, Iachina M., Skytthe A., Frederiksen H. 2015. Genetic and environmental influences in Dupuytren's disease: a study of 30,330 Danish twin pairs. J Hand Surg. 40:171–176. 10.1177/1753193414535720 PMC4810018 24835475

[msad130-B16] Lee SH , Wray NR, Goddard ME, Visscher PM. 2011. Estimating missing heritability for disease from genome-wide association studies. Am J Hum Genet. 88:294–305. 21376301 10.1016/j.ajhg.2011.02.002 PMC3059431

[msad130-B17] Li YI , Knowles DA, Humphrey J, Barbeira AN, Dickinson SP, Im HK, Pritchard JK. 2018. Annotation-free quantification of RNA splicing using LeafCutter. Nat Genet. 50:151–158. 29229983 10.1038/s41588-017-0004-9 PMC5742080

[msad130-B18] Lonsdale J , Thomas J, Salvatore M, Phillips R, Lo E, Shad S, Hasz R, Walters G, Garcia F, Young N, et al 2013. The genotype-tissue expression (GTEx) project. Nat Genet. 45:580–585. 23715323 10.1038/ng.2653 PMC4010069

[msad130-B19] Mafessoni F , Grote S, de Filippo C, Slon V, Kolobova KA, Viola B, Markin SV, Chintalapati M, Peyrégne S, Skov L, et al 2020. A high-coverage Neandertal genome from Chagyrskaya cave. Proc Natl Acad Sci U S A. 117:15132–15136. 32546518 10.1073/pnas.2004944117 PMC7334501

[msad130-B20] McLaren W , Gil L, Hunt SE, Riat HS, Ritchie GRS, Thormann A, Flicek P, Cunningham F. 2016. The ensembl variant effect predictor. Genome Biol. 17:122. 27268795 10.1186/s13059-016-0974-4 PMC4893825

[msad130-B21] Meyer M , Kircher M, Gansauge MT, Li H, Racimo F, Mallick S, Schraiber JG, Jay F, Prüfer K, de Filippo C, et al 2012. A high-coverage genome sequence from an archaic Denisovan individual. Science 338:222–226. 22936568 10.1126/science.1224344 PMC3617501

[msad130-B22] Mustafa KN , Khader YS, Bsoul AK, Ajlouni K. 2016. Musculoskeletal disorders of the hand in type 2 diabetes mellitus: prevalence and its associated factors. Int J Rheum Dis. 19:730–735. 26259148 10.1111/1756-185X.12617

[msad130-B23] Ng M , Lawson DJ, Winney B, Furniss D. 2019. Is Dupuytren's disease really a ‘disease of the Vikings’? J Hand Surg Eur. 45:273–279. 10.1177/1753193419882851 31663799

[msad130-B24] Ng M , Thakkar D, Southam L, Werker P, Ophoff R, Becker K, Nothnagel M, Franke A, Nürnberg P, Espirito-Santo AI, et al 2017. A genome-wide association study of Dupuytren disease reveals 17 additional variants implicated in fibrosis. Am J Hum Genet. 101:417–42. 28886342 10.1016/j.ajhg.2017.08.006 PMC5591021

[msad130-B25] Paigen K , Petkov PM. 2018. PRDM9 and its role in genetic recombination. Trends Genet. 34:291–300. 29366606 10.1016/j.tig.2017.12.017 PMC5878713

[msad130-B26] Prüfer K , de Filippo C, Grote S, Mafessoni F, Korlević P, Hajdinjak M, Vernot B, Skov L, Hsieh P, Peyrégne S, et al 2017. A high-coverage neandertal genome from Vindija cave in Croatia. Science 358:655–658. 28982794 10.1126/science.aao1887 PMC6185897

[msad130-B27] Prüfer K , Racimo F, Patterson N, Jay F, Sankararaman S, Sawyer S, Heinze A, Renaud G, Sudmant PH, de Filippo C, et al 2014. The complete genome sequence of a Neanderthal from the Altai mountains. Nature 505:43–49. 24352235 10.1038/nature12886 PMC4031459

[msad130-B28] Pruim RJ , Welch RP, Sanna S, Teslovich TM, Chines PS, Gliedt TP, Boehnke M, Abecasis GR, Willer CJ. 2010. Locuszoom: regional visualization of genome-wide association scan results. Bioinformatics 26:2336–2337. 20634204 10.1093/bioinformatics/btq419 PMC2935401

[msad130-B29] Racimo F , Marnetto D, Huerta-Sánchez E. 2017. Signatures of archaic adaptive introgression in present-day human populations. Mol Biol Evol. 34:296–317. 27756828 10.1093/molbev/msw216 PMC5400396

[msad130-B30] Rentzsch P , Witten D, Cooper GM, Shendure J, Kircher M. 2019. CADD: predicting the deleteriousness of variants throughout the human genome. Nucleic Acids Res. 47:D886–D894. 30371827 10.1093/nar/gky1016 PMC6323892

[msad130-B31] Rydberg M , Zimmerman M, Löfgren JP, Gottsäter A, Nilsson PM, Melander O, Dahlin LB, et al 2021. Metabolic factors and the risk of Dupuytren's disease: data from 30,000 individuals followed for over 20 years. Sci Rep. 11:14669. 34282190 10.1038/s41598-021-94025-7 PMC8289914

[msad130-B32] Saboeiro AP , Pokorny JJ, Shehadi SI, Virgo KS, Johnson FE. 2000. Racial distribution of Dupuytren's Ddsease in department of veterans affairs patients. Plast Reconstr Surg. 106:71–75. 10883614 10.1097/00006534-200007000-00013

[msad130-B33] Salari N , Heydari M, Hassanabadi M, Kazeminia M, Farshchian N, Niaparast M, Solaymaninasab Y, Mohammadi M, Shohaimi S, Daneshkhah A. 2020. The worldwide prevalence of the Dupuytren disease: a comprehensive systematic review and meta-analysis. J Orthop Surg Res. 15(1):495. 10.1186/s13018-020-01999-7 PMC7594412 33115483

[msad130-B34] Sankararaman S , Patterson N, Li H, Pääbo S, Reich D. 2012. The date of interbreeding between Neandertals and modern humans. PLoS Genet. 8:e1002947. 23055938 10.1371/journal.pgen.1002947 PMC3464203

[msad130-B35] Sjödin P , McKenna J, Jakobsson M. 2021. Estimating divergence times from DNA sequences. Genetics 217:iyab008. 33769498 10.1093/genetics/iyab008 PMC8049563

[msad130-B36] Staats KA , Wu T, Gan BS, O’Gorman DB, Ophoff RA. 2016. Dupuytren's disease susceptibility gene, EPDR1, is involved in myofibroblast contractility. J Dermatol Sci. 83:131–137. 27245865 10.1016/j.jdermsci.2016.04.015

[msad130-B37] ten Dam E-JPM , van Beuge MM, Bank RA, Werker PMN. 2016. Further evidence of the involvement of the Wnt signaling pathway in Dupuytren's disease. J Cell Commun Signal. 10:33–40. 26635199 10.1007/s12079-015-0312-8 PMC4850140

[msad130-B38] Võsa U , Claringbould A, Westra H-J, Bonder MJ, Deelen P, Zeng B, Kirsten H, Saha A, Kreuzhuber R, Yazar S, et al 2021. Large-scale cis- and trans-eQTL analyses identify thousands of genetic loci and polygenic scores that regulate blood gene expression. Nat Genet. 53:1300–1310. 34475573 10.1038/s41588-021-00913-z PMC8432599

[msad130-B39] Wei Y , Xiong ZJ, Li J, Zou C, Cairo CW, Klassen JS, Privé GG. 2019. Crystal structures of human lysosomal EPDR1 reveal homology with the superfamily of bacterial lipoprotein transporters. Commun Biol. 2:52. 30729188 10.1038/s42003-018-0262-9 PMC6363788

[msad130-B40] Zawistowski M , Fritsche LG, Pandit A, Vanderwerff B, Patil S, Schmidt EM, VandeHaar P, Brummett CM, Keterpal S, Zhou X, et al 2023. The Michigan Genomics Initiative: A biobank linking genotypes and electronic clinical records in Michigan Medicine patients. *Cell Genom.* 3(2):100257. 36819667 10.1016/j.xgen.2023.100257 PMC9932985

[msad130-B41] Zeberg H , Dannemann M, Sahlholm K, Tsuo K, Maricic T, Wiebe V, Hevers W, Robinson HPC, Kelso J, Pääbo S. 2020a. A Neanderthal sodium channel increases pain sensitivity in present-day humans. Curr Biol. 30:3465–3469.e4. 32707058 10.1016/j.cub.2020.06.045

[msad130-B42] Zeberg H , Kelso J, Pääbo S. 2020b. The Neandertal progesterone receptor. Mol Biol Evol. 37:2655–2660. 32437543 10.1093/molbev/msaa119 PMC7475037

[msad130-B43] Zerbino DR , Wilder SP, Johnson N, Juettemann T, Flicek PR. 2015. The ensembl regulatory build. Genome Biol. 16:56. 25887522 10.1186/s13059-015-0621-5 PMC4407537

[msad130-B44] Zhou Y , Browning BL, Browning SR. 2020. Population-specific recombination maps from segments of identity by descent. Am J Hum Genet. 107:137–148. 32533945 10.1016/j.ajhg.2020.05.016 PMC7332656

