# Supplementary material for: Major Genetic Risk Factors for Dupuytren's Disease Are Inherited From Neandertals
Source: Mol Biol Evol. 2023 Jun 14;40(6):msad130. doi: 10.1093/molbev/msad130 (PMC10266526; doi:10.1093/molbev/msad130)
Supplement: msad130_Supplementary_Data [file msad130_supplementary_data.zip › Supplementary Table 5.docx]

| **Chromosome** | 7 | 7 |
| --- | --- | --- |
| **Position** | 37,937,647 | 37,887,948 |
| **rsID** | rs2598104 | rs6462793 |
| **Reference allele** | T | G |
| **Alternative allele** | C | C |
| **GWAS P value (-log_10_)** | 84.3 | 36.5 |
| **OR [95% CI]** | 1.40 [1.35-1.45] | 1.27 [1.22-1.31] |
| **AAF** | 0.33 | 0.27 |
| **GTEx effect** | ↓ | N/A |
| **NES muscle (GTEx)** | -0.11 |  |
| **P value (GTEx)** | 1.0 x 10^-9^ |  |
| **NES fibroblasts (GTEx)** | -0.41 |  |
| **P value (GTEx)** | 5.3 x 10^-18^ |  |
| **eQTLgen effect** | ↓ | ↓ |
| **P value (eQTLgen)** | 3.3 x 10^-310^ | 1.9 x 10^-98^ |
