# Supplementary figures and images for: Major Genetic Risk Factors for Dupuytren's Disease Are Inherited From Neandertals
Source: Mol Biol Evol. 2023 Jun 14;40(6):msad130. doi: 10.1093/molbev/msad130 (PMC10266526; doi:10.1093/molbev/msad130)

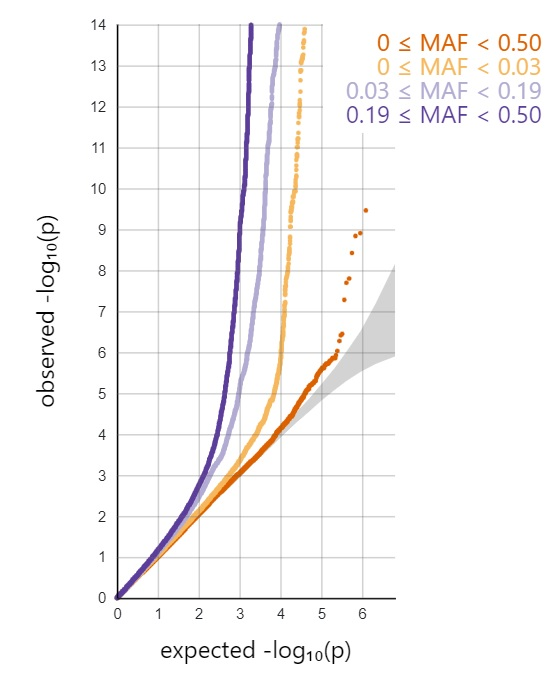

Supplement: msad130_Supplementary_Data [file msad130_supplementary_data.zip › Supplementary Figure 1.tif]

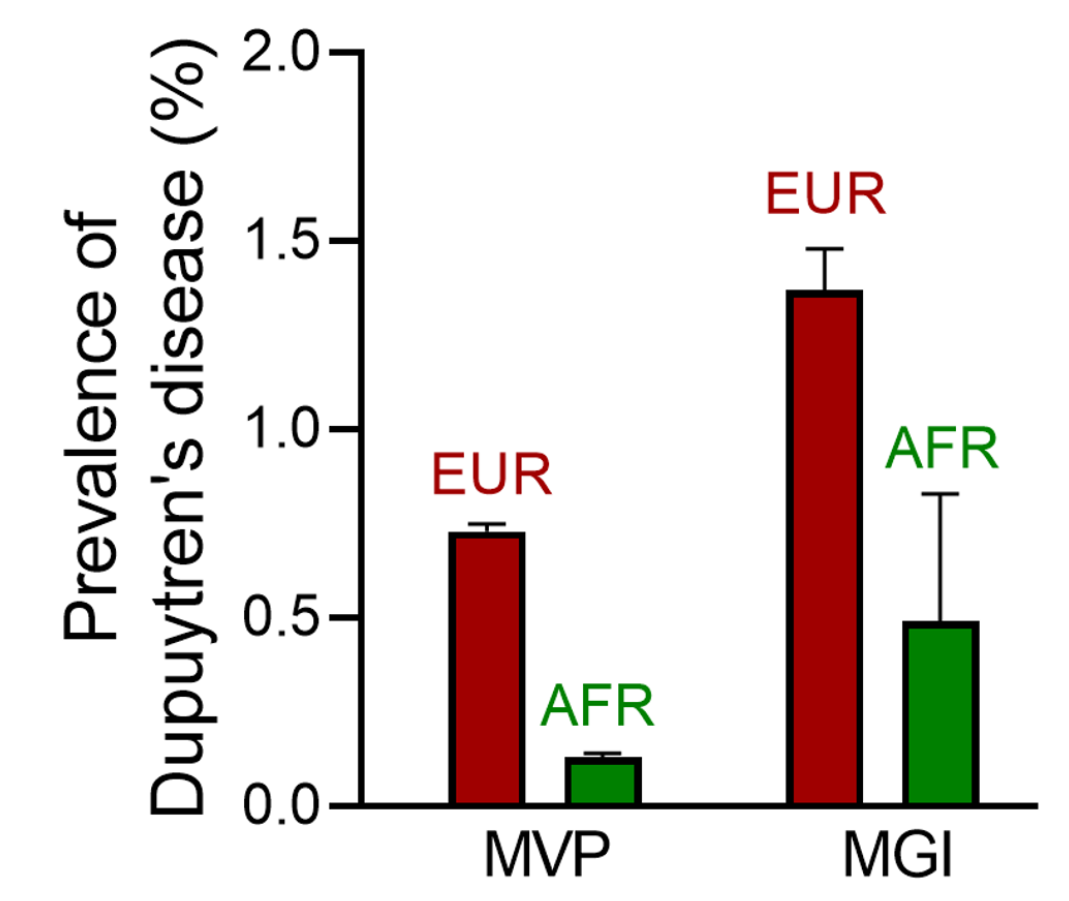

Supplement: msad130_Supplementary_Data [file msad130_supplementary_data.zip › Supplementary Figure 2.tif]

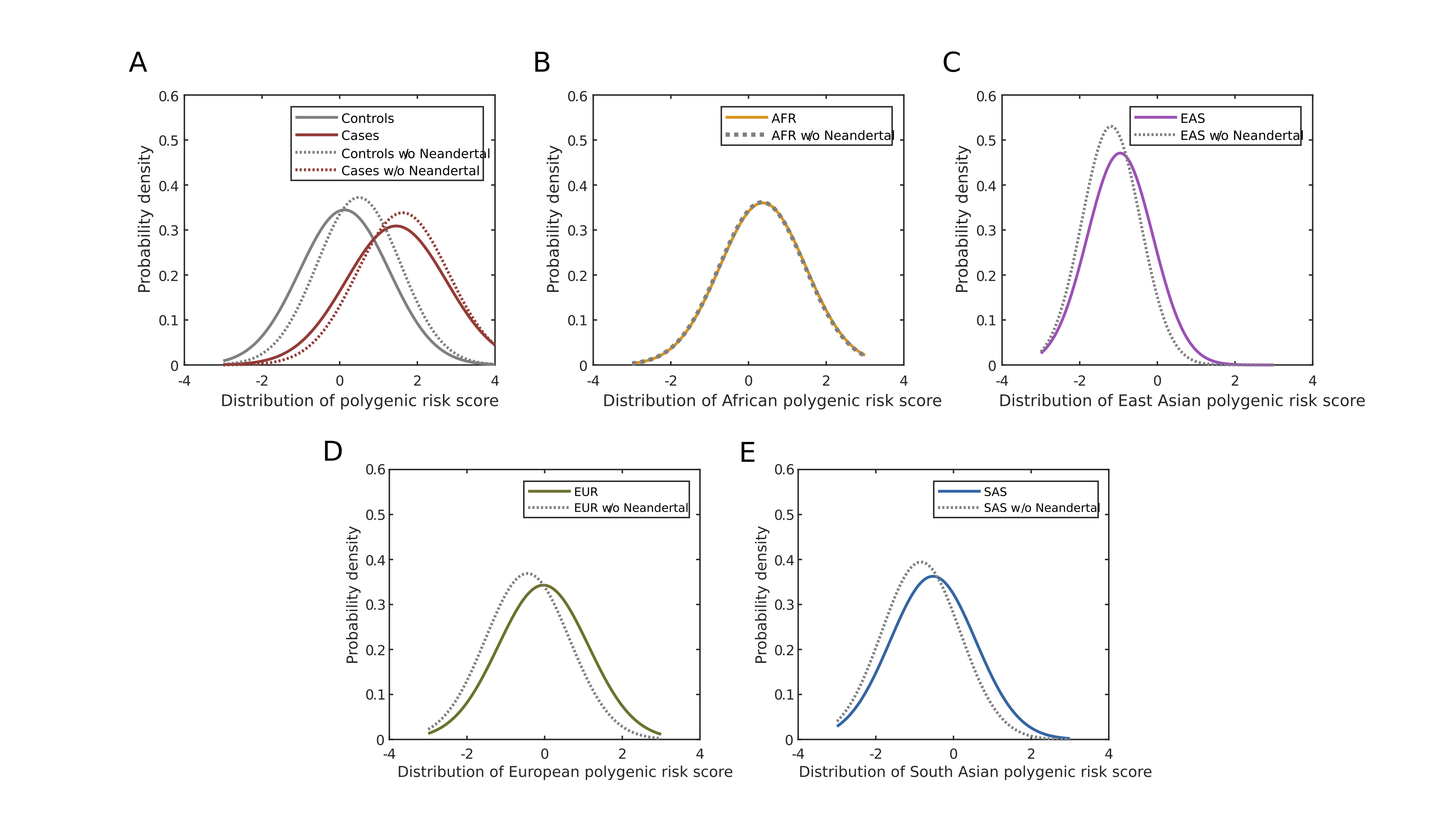

Supplement: msad130_Supplementary_Data [file msad130_supplementary_data.zip › Supplementary Figure 3.tif]
